# A Maternal and Postnatal Ad Libitum Propionic Acid-Rich Diet in Mice Alters Intestinal Glia Proliferation and Inflammatory Response: Contrary to Effect in the Brain

**DOI:** 10.3390/ijms26199295

**Published:** 2025-09-23

**Authors:** Piotr P. Lagod, Latifa S. Abdelli, Saleh A. Naser

**Affiliations:** 1 Burnett School of Biomedical Sciences, College of Medicine, University of Central Florida, Orlando, FL 32816, USA; piotr.lagod@ucf.edu; 2 Health Sciences Department, College of Health Professions and Sciences, University of Central Florida, Orlando, FL 32816, USA; latifa.abdelli@ucf.edu

**Keywords:** autism, ASD, propionic acid, GI comorbidities, gliosis

## Abstract

We previously demonstrated that propionic acid (PPA), a common food preservative and a metabolic byproduct of bacteria in dysbiosis (as seen in individuals affected with autism spectrum disorder, ASD), can lead to increased brain astrocyte proliferation and neuroinflammation in mice. We also showed that in vitro treatment of human neural stem cells with PPA increased glial cell vs. neuron differentiation and yielded a marked increase in pro-inflammatory cytokines. In this study, a group of mice (FVB/N-Tg(GFAPGFP)14Mes/J) was exposed in utero through the maternal diet and postnatally ad libitum to a PPA-rich diet, while the control group was fed a normal diet. Intestinal tissue from offspring mice at 1 month (1 M) and 5 months (5 M) were then studied for neurodifferentiation and gastrointestinal (GI) inflammation. There was a significant increase in *GFAP* (Glial fibrillary acidic protein) expression (1.5427-fold and 1.6097-fold in 1 M and 5 M, respectively) and GFAP protein levels (1.5616-fold and 1.6701-fold in 1 M and 5 M, respectively) in the PPA group mice. It is worth mentioning that the expression level of intestinal astrocyte markers in the PPA group was significantly and multi-fold lower than that in the brain tissue. Contrary to data from brain tissue, the expression of pro-inflammatory cytokines in the PPA group decreased in intestinal tissue at 5 M (*IL-6*: 0.4403-fold; *TNF-α*: 0.4007-fold), while IL-10 expression and protein levels increased (1.9360-fold and 1.3428-fold, respectively). The data demonstrates that although there was a significant increase in GFAP in the intestine suggesting gliosis, there was an overall anti-inflammatory cytokine profile. The effect of PPA on intestinal cytokines is most likely in part due to the lower expression of GFAP in the enteric nervous system than the central nervous system (and the lower number of intestinal glia than astrocytes in the brain) and the dominance of intestinal macrophages and other immune cells compared to that in the brain. The overall finding strongly suggests that the PPA-rich diet affects the enteric glia state as shown by an increase in GFAP; however, it maintains the overall anti-inflammatory cytokine profile, possibly due to M2 macrophage polarization.

## 1. Introduction

The gut microbiome and its metabolites were implicated in a plethora of diseases in recent years, including autism spectrum disorder (ASD) [1,2]. The gastrointestinal (GI) tract harbors bacteria (1000 species), fungi, and archaea [3], in which the combined number of genes present in those microorganisms is approximately 100- to 150-fold higher than that of the human host [4,5,6]. Microbial metabolites exert a large influence on the host, not only directly on the GI tract, but also on the entire organism, including the nervous system [7]. The microbiota and its host have a symbiotic relationship, in which the former is an important mediator of homeostasis. This includes regulation of the metabolism, immune response, brain function, and intestinal permeability. It also aids in digestion and in the defense against pathogens [7,8,9]. However, an imbalance in the microbial composition (dysbiosis) [10] was associated with many neurological diseases, including ASD [1,11].

ASD is a neurodevelopmental disorder that manifests itself through communication, social, and behavioral difficulties (which includes restrictive and repetitive behavior), and potential cognitive impairments [12]. According to the newest edition of the Diagnostic and Statistical Manual of Mental Disorders—5th edition (DSM-5), ASD encompasses autistic disorder, pervasive developmental disorder not otherwise specified (PDD-NOS), Asperger’s disorder, and childhood disintegrative disorder [13,14]. The prevalence of ASD quadrupled in the last 20 years and now affects 1 in 36 children (8 years old) [12,15]. Currently, there is a lack of effective treatment for ASD, and behavioral therapies aiming to diminish ASD characteristics and increase patients’ independence are costly and time-consuming for both patients and caregivers [16,17]. A significant amount of research points to the complex interaction between genetic and environmental factors as the etiology of ASD, where in recent years propionic acid (PPA) has gained notoriety as one of the environmental factors [12,18].

PPA is a short chain fatty acid (SCFA) with 3 carbons in its structure. PPA is present in the human gut, as it is released by certain bacteria as a product of the fermentation of undigested carbohydrates and, to a smaller extent, peptides [19,20]. Besides PPA, other commonly present SCFAs in the human gut are acetic acid (AA) and butyric acid (BA), constituting a total concentration of 50–200 mM in the large intestine [21]. Mounting evidence shows that ASD patients and their mothers have an increased number of bacteria producing large quantities of PPA, especially *Clostridium*, *Bacteroides*, and *Desulfovibrio* [18,22,23,24,25,26]. Several studies also showed that the level of PPA is significantly elevated in the blood and stool of ASD patients [27,28,29]. Besides PPA being produced by intestinal bacteria, it may also be ingested, as PPA and its salts are a commonly used food preservative due to their anti-bacterial and anti-fungal properties [30,31]. Additionally, children with propionic acidemia (a genetic disease in which the propionyl-CoA carboxylase enzyme has diminished activity due to a mutation and is characterized by a high level of PPA in circulation) exhibit ASD-like symptoms [32]. Finally, propionic acid injections were shown to invoke ASD-like behavior and neuroinflammation in rat models [33,34,35,36].

GI disorder symptoms are 3 to 4 times more common in children with ASD than in the general population, with a significant increase in the rates of constipation, diarrhea, and abdominal pain [37,38]. It is plausible that the genetic and environmental factors contributing to the core ASD symptoms in the CNS could also affect the development of the enteric nervous system (ENS) [39]. The ENS is an essential part of the GI tract, as it regulates diverse processes in the GI such as intestinal motility, secretion of various enzymes, intestinal barrier maintenance, nutrient detection, and to a large extent, immunological defense [40,41,42]. The ENS comprises enteric neurons and enteric glia, with the latter significantly outnumbering enteric neurons [43,44]. They both originate from neural crest cells and populate the developing gut during the embryonic stage. Enteric glia and neurons are organized into ganglia, which in turn, are organized into two distinctive plexuses: submucosal (Meissner’s) and myenteric (Auerbach’s) [45,46]. The former plexus is sandwiched between the muscularis submucosa and circular muscle, while the latter is located between the circular and longitudinal muscle layers [44]. While the submucosal plexus orchestrates vasodilation, nutrient absorption, and secretion, the myenteric plexus is responsible for the regulation of GI motility by sending signals to the circular and longitudinal muscles [44,46,47,48].

Intestinal glia exhibit remarkable morphological and functional resemblance to astrocytes in the CNS. They also share common markers such as the glial fibrillary acidic protein (GFAP) and S100β [43]. GFAP was found to be elevated in the blood and brain of ASD patients in several studies [49,50,51]. An increase in GFAP expression suggests a state of “gliosis,” where glia cells are activated, proliferate, and play a role in immune modulation [43,52]. Besides intestinal glia, other specialized immune cells play a crucial role in the modulation of the immune response; these include macrophages, T cells, dendritic cells, and others [53,54].

Our laboratory previously reported that in vitro treatment of human fetal-derived neural stem cells (NSCs, StemPro Neural Stem Cells, Thermo Fisher Scientific, Waltham, MA, USA, A15654) with propionic acid salt at 2 μM concentration significantly shifted NSC differentiation, with an increase towards glial cells (expressing GFAP) and a decrease towards neuronal cells. This shift was also associated with an increase in pro-inflammatory cytokines [55]. Using a mouse model, we also demonstrated that a PPA-rich diet led to glia cell over-proliferation and neuroinflammation in the brain [56]. However, the effect of PPA on intestinal tissue in mice has not been studied.

Taking into consideration our previous findings in the in vitro model and mouse study focusing on PPA’s effect on the CNS, we hypothesize that a PPA-rich diet will alter intestinal glia proliferation and cytokine response.

In this study, we exposed transgenic mice (FVB/N-Tg(GFAPGFP)14Mes/J) overexpressing GFP under the GFAP promoter to a PPA-rich diet, both during their embryonic development and after weaning. We assessed the expression and protein levels of the markers for enteric glia and neuronal cells and the effects on cytokines in the intestine (jejunum). We chose to examine the IL-6 and TNF-α cytokines as they are found to be elevated in the plasma of ASD patients, while IL-10 was found to be diminished [57,58,59,60]. We also examined the levels of *iNOS* and *CD206*, which in conjunction with the cytokine profile would provide insight into the tissue’s inflammatory environment. Lastly, we exposed THP-1 monocyte-derived macrophages to PPA in the in vitro cell culture to determine its effect on macrophages alone, without interference from other cell types.

## 2. Results

### 2.1. GFAP Abundance in the Intestine Was Significnalty Elevated in Offspring Mice in the PPA-Rich Diet Group Based on the Fluorescence from the GFAP-GFP Construct

**Figure 1:**
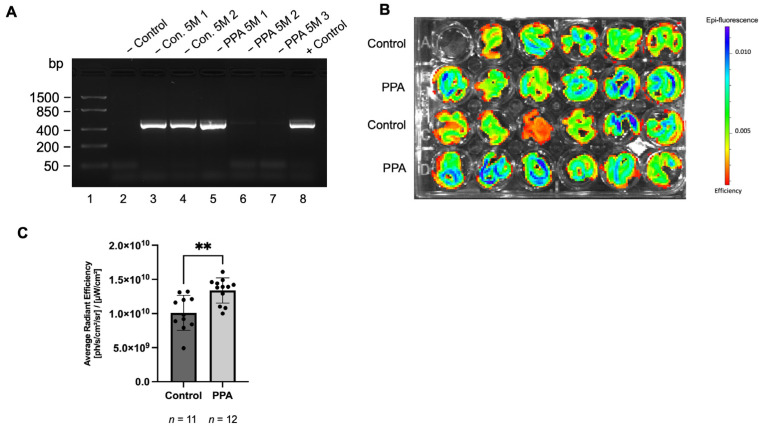
Fluorescence in the mice intestine GFAP-GFP construct. Subfigure (**A**) consists of a representative image of agarose gel with the PCR product. The first lane contains the DNA ruler, while lanes 2 and 8 contain the negative and positive controls, respectively. Subfigure (**B**) contains a raw image of the epi-fluorescence from the GFAP-GFP construct. The scale is included in the picture, with higher epi-fluorescence indicated by a higher amount of blue color in the image. Subfigure (**C**) represents the measured average radiant efficiency expressed in [ph/s/cm^2^/sr]/[µW/cm^2^] (where “ph” stands for photons and “sr” for steradian). Data is presented as mean ± SD with *n* number indicated below the graph. A *p*-value < 0.01 is considered significant (**).

Fluorescence levels from the glia marker GFAP-GFP construct were measured to determine if GFAP was overexpressed in the intestine (jejunum) of mice exposed to PPA. Prior to fluorescence measurement, the presence of the GFAP-GFP construct was verified by obtaining DNA from the tail snips, which was subsequently subjected to PCR with primers specific to the GFAP-GFP construct. Representative results are presented in Figure 1A, where the animals designated as Con. 5 M 1, Con. 5 M 2, and PPA 5 M 1 were positive for the construct and, thus, included in the fluorescence measurement. Alternatively, the animals designated as PPA 5 M 2 and PPA 5 M 3 were negative for the construct and, thus, omitted from the measurements. Figure 1B shows the epi-fluorescence of the intestine at 5 months (5 M), where the colors that are closer to blue in the spectrum signify higher epi-fluorescence. The average fluorescence (Figure 1C) was significantly elevated in the PPA group with a *p*-value < 0.01 (**, *n* = 11 in the control and *n* = 12 in the PPA group). In the control, the mean radiant efficiency was 1.0098 × 10^10^ (±2.4414 × 10^9^) [ph/s/cm^2^/sr]/[µW/cm^2^], while in the PPA group it was 1.3361 × 10^10^ (±1.7768 × 10^9^) [ph/s/cm^2^/sr]/[µW/cm^2^].

### 2.2. GFAP Increased in the PPA-Diet Mice Group with No Change in the Neuronal Marker Tubulin IIIβ

**Figure 2:**
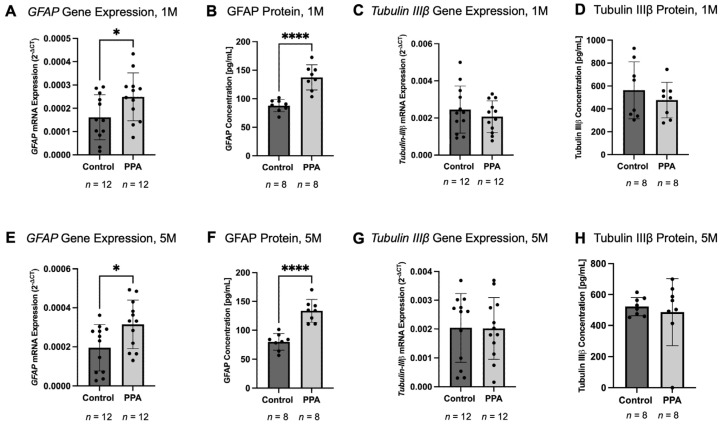
Measurement of the expression and protein concentration of enteric glia and neuronal markers (GFAP and Tubulin IIIβ, respectively) in the mice intestine. Subfigures (**A**,**E**) represent *GFAP* gene expression (in the 1 M and 5 M studies, respectively), while subfigures (**C**,**G**) represent *Tubulin IIIβ* gene expression. Gene expression results were based on qPCR. Data is represented as relative gene expression using the 2^−∆CT^ method. Subfigures (**B**,**F**) represent GFAP concentration (pg/mL) at 1 M and 5 M, while subfigures (**D**,**H**) represent Tubulin IIIβ protein concentration (ng/mL) in the mice intestine. Data is presented as mean ± SD with the *n* number indicated below each graph. (**A**) *p*-value < 0.05 (*) and *p* < 0.0001 (****) are considered significant.

We further validated our fluorescence results for GFAP-GFP by the quantification of the gene expression and protein concentration of GFAP. We also assessed the gene expression and protein concentration of the neuronal marker Tubulin IIIβ by utilizing qPCR and ELISA, respectively. The measurements were conducted at both 1 month (1 M) and 5 months (5 M), in which the animals were considered adolescents and adults, respectively. *GFAP* gene expression was significantly elevated in the PPA groups from both the 1 M and 5 M animals (*p*-value < 0.05 (*), *n* = 12). The relative expression of *GFAP* in the 1 M control group (based on the 2^−∆CT^ method) was 1.6138 × 10^−4^ (±9.678 × 10^−5^), while the expression in the PPA group was 2.4896 × 10^−4^ (±1.0297 × 10^−4^) and consisted of a 1.5427-fold increase (Figure 2A). In the 5 M study, *GFAP* relative expression was 1.9457 × 10^−4^ (±1.1841 × 10^−4^), while in the PPA group it was 3.1466 × 10^−4^ (±1.2448 × 10^−4^), which was a 1.6097 increase (Figure 2E). GFAP concentration was significantly elevated in the PPA group vs. the control (*p*-value < 0.0001 (****), *n* = 8) at both 1 M and 5 M. The protein concentration was 87.9537 (±10.7766) [pg/mL] in the control compared to 137.3518 (±22.0402) [pg/mL] in the PPA group; this was a 1.5616-fold increase (Figure 2B). Similarly, in the 5 M study, the GFAP concentration was 79.9676 (±14.3481) [pg/mL] in the control, compared to 133.5556 (±19.8290) [pg/mL] in the PPA group, which was considered a 1.6701-fold increase (Figure 2F). There was no significant difference in the gene expression or the protein concentration of Tubulin IIIβ in the 1 M and 5 M groups; however, a slightly decreasing trend was noted. At 1 M, the average gene expression of *Tubulin IIIβ* in the control group was 2.4532 × 10^−3^ (±1.2716 × 10^−3^), while in the PPA group it was 2.0723 × 10^−3^ (±8.5416 × 10^−4^) (Figure 2C). At 5 M, the average gene expression of *Tubulin IIIβ* in the control group was 2.0415 × 10^−3^ (±1.1918 × 10^−4^), while in the PPA group it was 2.0200 × 10^−3^ (±1.0729 × 10^−4^) (Figure 2G). The protein concentration of Tubulin IIIβ at 1 M was very similar in the control and PPA groups (0.5625 ± 0.2486 pg/mL and 0.4763 ± 0.1549 pg/mL, respectively) (Figure 2D). At 5 M, the mean protein concentration was 0.5220 ± 0.0593 pg/mL in the control and 0.5559 ± 0.0961 pg/mL in the PPA group (Figure 2H). *GFAP* and *Tubulin IIIβ* gene expression is also summarized in Table 1.

### 2.3. IL-6 and TNF-α Decreased, While IL-10 Increased in the Intestine of the PPA Group

**Figure 3:**
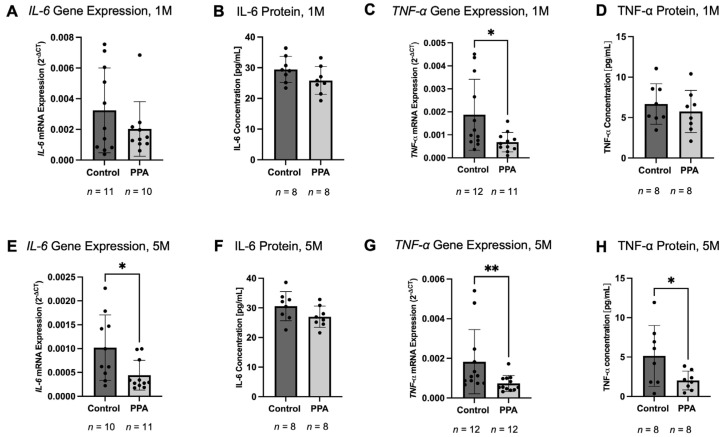
The expression and protein concentration of major pro-inflammatory cytokines: IL-6 and TNF-α. Subfigures (**A**,**E**) represent the expression of *IL-6* at 1 M and 5 M, respectively, while Subfigures (**C**,**G**) represent the expression of *TNF-α* at 1 M and 5 M, respectively. The gene expression data is presented as relative gene expression using the 2^−∆CT^ method. Subfigures (**B**,**F**) represent IL-6 cytokine concentration in the intestinal tissue lysate at 1 M and 5 M, respectively. Subfigures (**D**,**H**) represent TNF-α concentration in the intestinal tissue at 1 M and 5 M, respectively. Data is presented as mean ± SD with the *n* number indicated below each graph. A *p*-value < 0.05 (*) and *p* < 0.01 (**) are considered significant.

Intestinal homogenates were also tested for the expression and abundance of key pro-inflammatory and anti-inflammatory cytokines in the 1 M and 5 M groups. In the 1 M study, *IL-6* gene expression showed a decreasing trend (Figure 3A), where the expression of *IL-6* in the control was 3.2423 × 10^−3^ (±2.7657 × 10^−3^), while in the PPA group it was 2.0273 × 10^−3^ (±1.7819 × 10^−3^). In the 5 M study, there was a significant decrease in *IL-6* (*p*-value < 0.05 (*), *n* = 10 and *n* = 11 for the control and PPA group, respectively) (Figure 3E). In the control, *IL-6* expression was 1.0224 × 10^−3^ (±7.2737 × 10^−4^), while in the PPA group it was 4.5018 × 10^−4^ (±3.2824 × 10^−4^), which was a 0.4403-fold decrease. At the protein levels, IL-6 showed a decreasing trend at both 1 M and 5 M (Figure 3B,F, respectively). At 1 M, the mean IL-6 level in the control was 29.4604 (±4.2701) [pg/mL], while in the PPA group it was 25.8571 (±4.5213) [pg/mL]. At 5 M, a similar trend was seen, where the mean concentration of IL-6 was equal to 30.5957 (±4.8989) [pg/mL] in the control and 26.9860 (±3.5853) [pg/mL] in the PPA group. The gene expression of another key cytokine, *TNF-α*, was significantly decreased at 1 M in the PPA group (*p*-value < 0.05 (*), *n* = 12 and *n* = 11 for the control and PPA group, respectively) (Figure 3C). In the control group, the gene expression was 1.8724 × 10^−3^ (±1.5407 × 10^−3^), while in the PPA group it was 6.8313 × 10^−4^ (±4.2450 × 10^−4^), as shown in Figure 3C. In the 5 M study, *TNF-α* gene expression was also significantly decreased in the PPA group vs. the control, as depicted in Figure 3G (*p*-value < 0.01 (**), *n* = 12). In the control, the mean gene expression was equal to 1.8280 × 10^−3^ (±1.6179 × 10^−3^), while in the PPA group it was equal to 7.3261 × 10^−4^ (±4.0197 × 10^−4^), which was a 0.4007-fold decrease. TNF-α protein concentration at 1 M showed a decreasing trend, with a mean concentration of 6.6812 (±2.5135) [pg/mL] in the control and 5.7551 (±2.6180) [pg/mL] in the PPA group (Figure 3D). In the 5 M study, TNF-α protein concentration in the intestine was significantly decreased in the PPA vs. control group (*p*-value < 0.01 (**), *n* = 8). In the control group, the protein concentration was 5.1410 (±2.0160) [pg/mL], while in the PPA group it was 3.860 (±1.1985) [pg/mL], which was a 0.3921-fold decrease (Figure 3H).

**Figure 4:**
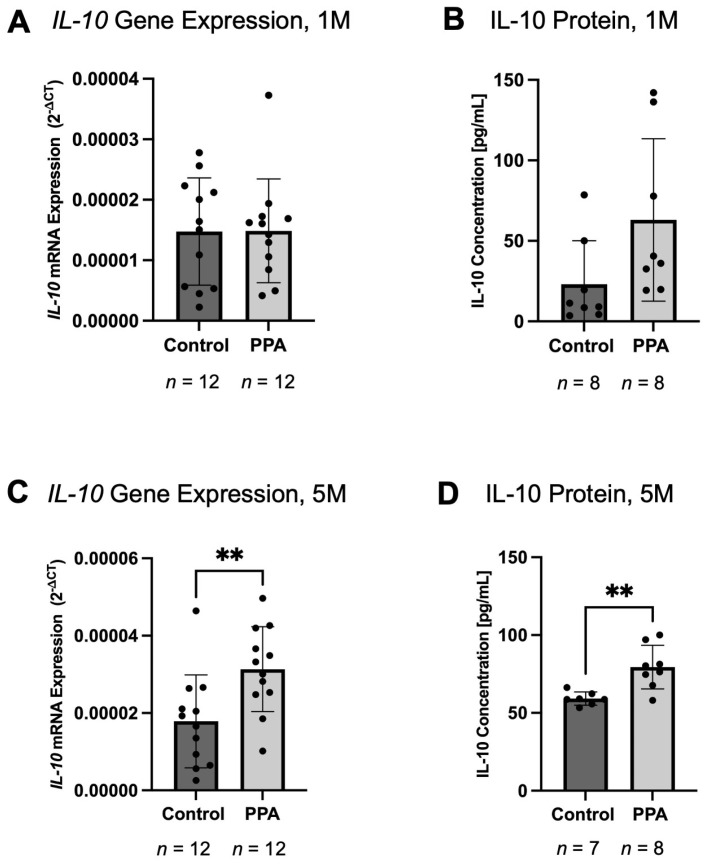
The expression and protein concentration of the major anti-inflammatory cytokine IL-10. Subfigures (**A**,**C**) represent the expression of *IL-10* at 1 M and 5 M, respectively. The gene expression data is presented as relative gene expression using the 2^−∆CT^ method. Subfigures (**B**,**D**) represent IL-10 concentration at 1 M and 5 M, respectively. Data is presented as mean ± SD with the *n* number indicated below each graph. A *p*-value < 0.01 (**) is considered significant.

As opposed to the pro-inflammatory cytokines, the gene expression and protein concentration of *IL-10* (an anti-inflammatory cytokine) was significantly increased in the 5 M study (*p*-value < 0.01 (**)) and showed an increasing trend at 1 M. The gene expression of *IL-10* at 1 M was unchanged (Figure 4A) in the 1 M study. However, IL-10 protein concentration showed an increasing trend (Figure 4B), with a mean concentration of 23.0625 (±26.9857) pg/mL in the control group and 63.7317 (±50.3903) pg/mL in the PPA group (*n* = 8). In the 5 M study, *IL-10* gene expression in the control group was 1.7865 × 10^−5^ (±1.2024 × 10^−5^) and 3.4585 × 10^−5^ (±1.3224 × 10^−5^) in the PPA group, which was a 1.9360-fold increase (*n* = 12) (Figure 4C). At 5 M, IL-10 protein concentration was 59.1429 (±4.2464) [pg/mL] in the control group and 79.4167 (±13.9702) [pg/mL] in the PPA group, which was a 1.3428-fold increase (*n* = 7 in the control and *n* = 8 in the PPA group) (Figure 4D). *IL-6*, *TNF-α*, and *IL-10* gene expression is also summarized in Table 1.

### 2.4. Pro-Inflammatory Macrophage (iNOS) Was Decreased in the PPA Group, While Anti-Inflammatory Macrophage (CD206) Was Increased

**Figure 5:**
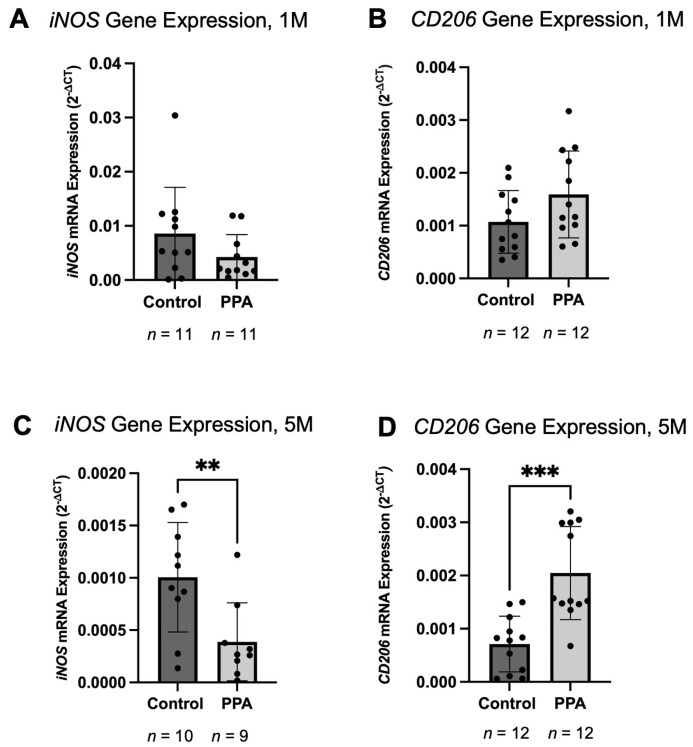
The expression of markers for pro-inflammatory (M1) and anti-inflammatory (M2) macrophages: *CD206* and *iNOS*, respectively. Subfigures (**A**,**C**) represent the expression of *iNOS* at 1 M and 5 M, respectively, while Subfigures (**B**,**D**) represent the expression of *CD206* at 1 M and 5 M, respectively. The gene expression data is presented as relative gene expression using the 2^−∆CT^ method. Data is presented as mean ± SD with the *n* number indicated below each graph. A *p*-value < 0.01 (**) and *p*-value < 0.001 (***) are considered significant.

To determine if a PPA-rich diet affected the polarization of intestinal macrophages, we measured the expression of *iNOS* and *CD206*, which are markers for M1 pro-inflammatory macrophages and M2 anti-inflammatory macrophages, respectively. At 1 M, the expression of *iNOS* showed a decreasing trend, with a mean expression of 8.5820 × 10^−3^ (±8.5385 × 10^−3^) in the control group and 4.2468 × 10^−3^ (±4.1335 × 10^−3^) in the PPA group (Figure 5A). At 5 M (Figure 5C), *iNOS* expression was significantly decreased (*p*-value < 0.01 (**), *n* = 10 in the control and *n* = 9 in the PPA group). In the control group, the mean expression was 1.0053 × 10^−3^ (±5.2361 × 10^−4^), whereas in the PPA group, it was 3.8800 × 10^−4^ (±3.7267 × 10^−4^), which was a 0.3858-fold decrease. The expression of *CD206* in the 1 M study (Figure 5B) showed an increasing trend (*n* = 12), with a mean expression of 1.0722 × 10^−3^ (±5.9261 × 10^−4^) in the control group and 1.5913 × 10^−3^ (±8.2216 × 10^−4^) in the PPA group, which was a 1.4841-fold increase. In the 5 M study (Figure 5D), there was a significant increase in CD206 expression (*p*-value < 0.001 (**), *n* = 12), with a mean expression of 7.1255 × 10^−4^ (±5.2349 × 10^−4^) in the control group and 2.0468 × 10^−3^ (±8.7524 × 10^−4^) in the PPA group, which was a 2.872-fold increase.

### 2.5. The Gene Expression and Protein Levels of Pro-Inflammatory Cytokines in THP1 Macrophages Were Lower in the PPA-Treated Groups

**Figure 6:**
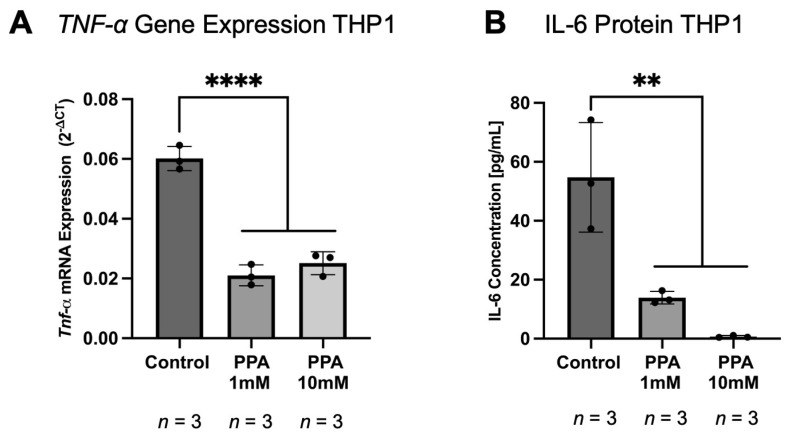
The gene expression of *TNF-α* and protein concentration of IL-6 in THP1 macrophages. Subfigure (**A**) represents relative gene expression of *TNF-α* using the 2^−∆CT^ method. Subfigure (**B**) represents IL-6 protein level measured with an ELISA kit. Data is presented as mean ± SD with the *n* number indicated below each graph. A *p*-value < 0.01 (**) and *p*-value < 0.0001 (****) are considered significant.

To further determine the effect of PPA on the immune cells, we exposed monocyte-derived THP1 macrophages to PPA (at 1 mM and 10 mM) and assessed the expression of *TNF-α* and the concentration of IL-6 with qPCR and ELISA, respectively. The expression of *TNF-α* was significantly decreased (*p*-value < 0.0001 (****), *n* = 3) in the PPA treatments (both at 1 mM and 10 mM; Figure 6A). The expression of *TNF-α* in the control was 6.0133 × 10^−2^ (±4.0397 × 10^−3^), while it was 2.1061 × 10^−2^ (±3.4839 × 10^−3^) at 1 mM PPA and 2.5136 × 10^−2^ (±3.8259 × 10^−3^) at 10 mM PPA. The protein concentration of IL-6 was also significantly reduced (*p*-value < 0.01 (**), *n* = 3; Figure 6B). In the control group, IL-6 concentration was 54.7446 (±18.5523) pg/mL, while it decreased to 13.9247 (±2.1346) pg/mL in 1 mM PPA and 0.6989 (±0.3746) pg/mL in 10 mM PPA.

### 2.6. A Comparison of the Gene Expression of Glial, Neuronal, and Inflammatory Markers Between the Brain and Intestine

To further emphasize the role of immune cells in creating an overall anti-inflammatory response in the intestine while exposed to a PPA-rich diet, the overall expression of glia, neuronal markers, and pro- and anti-inflammatory cytokines was compared between the brain and intestine (Table 1). The brain expression data was previously published [56]; however, it was presented as a fold change using the 2^−∆∆CT^ method. This table presents the gene expression in both the brain (previously published) and intestine using the 2^−∆CT^ method. The expression of *GFAP* in the brain is around 250-fold higher than in the intestinal sample. Similarly, the expression of *Tubulin IIIβ* is also much higher in the brain than in the intestine (50 to 80-fold higher). On the other hand, the expression of pro-inflammatory cytokines (*IL-6* and *TNF-α*) is comparable between the brain and intestine. The expression of *IL-10* was several folds lower in the intestine than the brain.

## 3. Discussion

Due to a staggering increase in ASD’s prevalence in the past decades [15,61] and a lack of sufficient treatments [55], there is a great need to increase efforts to better understand the etiology of ASD and devise possible means of its prevention and treatment. In many cases, ASD patients not only suffer from core ASD symptoms stemming from CNS, but also from GI comorbidities that impact the patients’ day-to-day lives [37,38]. The GI comorbidities could be associated with deficits in the ENS, as the ENS and CNS share many common characteristics [43]. A large body of research shows that ASD is associated with neuroinflammation [62,63,64] and elevated levels of GFAP [49,51], an astrocytic marker, which is also a marker for enteric glia. ASD is believed to stem from a complex set of genetic and environmental factors [18]. Our laboratory focuses on PPA, which could be one of the latter, as it was found that PPA is elevated in the stool of ASD patients. Additionally, bacterial species producing high levels of PPA were found to be elevated in the GI tract of ASD patients and their mothers [18]. Propionic acid is also present in many baked and packaged goods, especially those with a long shelf life, as PPA has anti-fungal properties [30].

Other studies showed that PPA injections in rats were associated with ASD-like symptoms and neuroinflammation [33,35,36,65,66]. Our lab previously showed that PPA can alter neural stem cell differentiation to favor glia cells vs. neurons and significantly increase pro-inflammatory cytokines [55]. Also, using an animal model, we show that PPA present in the maternal diet and the administration of PPA to offspring post-weaning leads to a significant increase in GFAP gene expression and protein levels in the brain of offspring, with a decreasing trend in the expression of neuronal markers. We also showed that pro-inflammatory cytokines (IL-6 and TNF-α) were increased in the brain of the offspring, while the anti-inflammatory cytokine IL-10 was decreased [56]. That study showed that a PPA-rich diet can induce glia cell over-proliferation/activation and neuroinflammation in the CNS. Intrigued by those findings, we designed this study to test if a similar effect of a PPA-rich diet could be observed in the ENS and possibly provide an explanation for the GI symptoms in ASD.

In this study, we exposed FVB/N-Tg(GFAPGFP)14Mes/J transgenic mice expressing GFP under the GFAP promoter to PPA during embryonic development via the maternal diet and directly via the diet fed to offspring after weaning. Experiments were conducted on 1-month-old mice (at the time of weaning) and 5-month-old mice, which corresponds to adolescent and adult mice, respectively. This was performed to better capture differences associated with the age of the animals and to see if prolonged and direct exposure to PPA via the diet would influence the experimental outcomes. The concentration of PPA in the diet was 5000 mg/kg of total food, as this is the highest allowable concentration of added PPA in the food industry [30]. Also, several studies utilized 250 mg/kg of body weight of PPA orally or 500 mg/kg as a subcutaneous injection [66,67]. In healthy individuals, the concentration of PPA in the proximal colon is 10–30 mM [20,68]. In the ASD patients, it was found that the PPA concentration was reaching 1.2 mmol per 1 kg of wet fecal weight, where the control had significantly lower levels [69]. Although the concentration of PPA used in this study is higher than what could be expected from a regular balanced diet, it helped to accommodate for PPA being produced by the dysbiotic gut, where PPA producers are elevated in ASD [18]. It also helped to account for a variable response to PPA due to the genetic predisposition seen in the human population; some individuals may be sensitive to a much lower dosage of PPA in the diet, while others would require a much higher dosage [55]. This would explain why some individuals may not develop ASD symptoms despite dysbiosis and a PPA-rich diet. We observed a significant elevation of GFAP based on the fluorescence from GFP-GFAP. To further validate our findings, we assessed GFAP gene expression and protein concentration in the intestinal sample. We found that GFAP was significantly elevated in both the 1 M and 5 M studies at both the gene expression and protein levels. After we established that glia markers in the intestine were elevated, which supported our hypothesis and matched our previous in vitro findings [55] and findings in the CNS of the mouse model [56], we assessed the levels of the pro- and anti-inflammatory cytokines in the intestine. Intriguingly, we found that at 1 M, the RNA and protein levels of both IL-6 and TNF-α showed a diminishing trend with reaching significance in the 5 M study. The anti-inflammatory cytokine IL-10 showed an increasing trend at 1 M, with a significant increase at the 5 M timepoint. Although GFAP was elevated in the intestine as often seen in gliosis [70], it was surprising that the pro-inflammatory cytokines were downregulated and anti-inflammatory cytokines were upregulated. This effect could possibly be caused by PPA exerting an anti-inflammatory effect on the intestinal immune cells, which includes intestinal macrophages [71]. Intestinal glia play a large role in immune modulation in the intestine [52,72]; however, intestinal glia cells are not abundant in the intestine as astrocytes are in the CNS. Although it is possible that intestinal glia cells could have a pro-inflammatory response to PPA similar to what we previously observed in vitro [55] and in the CNS [56], it is possible that their contribution to the inflammatory response was overpowered by the anti-inflammatory response from other cell types [73]. Indeed, we proceeded to assess the expression levels of *iNOS* and *CD206*, which are markers for M1 and M2 macrophages, respectively [74,75,76]. We found that at the 1 M timepoint, *iNOS* expression showed a decreasing trend, while *CD206* showed an increasing trend. At the 5 M timepoint, those findings became statistically significant. This observation is logical since the expression of GFAP in intestinal tissue in the PPA group was 250-fold less than that of the brain. Thus, the contribution of glia cells to the overall immune response can be overshadowed by macrophages and other immune cell types. However, those findings are preliminary and warrant further investigation involving immunostaining and colocalization studies as, although iNOS and CD206 are primarily markers for M1 and M2 macrophages, respectively, other cells can also express those markers [77,78,79]. We also tested the effect of PPA on THP-1 macrophages. We observed a decrease in IL-6 and TNF-α, which further supports the ant-inflammatory role of PPA on immune cells. Additional factors may be attributed to prolonged exposure to PPA in mice at the 5 M timepoint, as those animals were maintained on a propionic acid-rich diet after weaning. Additionally, our collaborators found that high levels of PPA in the diet alters the composition of gut microbiota, with an increase in taxa producing PPA, which could further exacerbate the effect of PPA in the diet [2].

ASD has a very complex etiology likely involving many genetic and environmental factors and interactions between them. Taking into account the comorbidities associated with ASD, especially those of the GI tract (which, with the ENS, could be considered the second brain in the gut [44]), this adds even more to the complexity of the issue. This brings some limitations to this study. First, the diet established for the mothers and then the offspring after weaning contained a high dosage of PPA, which would be difficult to obtain if compared to the human diet, as patients and their mothers are not expected to only consume highly processed food containing PPA. This dosage was chosen to account for the increase in PPA that could be produced by the bacteria in a dysbiotic gut, which is often seen in ASD patients, and for the genetic predisposition to PPA [18]. In future studies, a well monitored variable dosage (in different treatment groups) should also be considered, as in this study only one dosage premixed with food that was given ad libitum was administered. Additionally, the effects of PPA could have been exacerbated by the increase in PPA-producing bacteria in the PPA group, which was shown by our collaborators [2]. After weaning, the offspring were placed on the same diet as their mothers to mimic the GI microbiota transfer seen between ASD patients and their mothers [80]. However, due to this experimental design choice, we cannot determine if the effect of PPA that was seen could be evoked by a PPA-rich diet administered after weaning alone. Future studies, beyond the scope of this paper, could be envisioned, where the diet would be switched during weaning. In this setting, offspring from dams on a PPA-rich diet would be switched to a control diet and vice versa. In the envisioned study, behavioral assessment should also be included, as this study only focused on the physiological and molecular changes. Also, long term changes such as fibrosis and loss of function should also be assessed.

Short chain fatty acids, particularly PPA, could be seen as either a friend or a foe depending on the circumstances and the target organs. PPA was shown to be elevated in the blood and stool of ASD patients [18], and it was shown in several studies that it can evoke ASD-like symptoms and neuroinflammation in the CNS in rodent models [33,56]. In this study, our data showed that PPA-rich diet mice showed an increase in GFAP expression in the intestine, which is a sign of gliosis. This might bring insight on PPA’s effect in the intestine and a possibility of perturbation in the ENS, which is a promising avenue for further studies. The overall levels of pro-inflammatory cytokines were decreased, and the anti-inflammatory cytokines were increased, alongside alterations in iNOS and CD206, which suggests that PPA may also have an anti-inflammatory effect on immune cells; this was also confirmed by the in vitro experiment in the THP-1 cells. Overall, this study brings important insight to the role of PPA in the ENS and enteric gliosis.

## 4. Materials and Methods

### 4.1. Animal Maintenance and PPA Administration

All animal work and PPA administration was conducted in accordance with previously published protocol [56] and followed the University of Central Florida Institutional Animal Care and Use Committee (UCF-IACUC) guidelines (Animal Use Approval #: PROTO202000002). Mice used in this study were obtained from Jackson Laboratories (FVB/N-Tg(GFAPGFP)14Mes/J), JAX stock #003257, The Jackson Laboratory, Bar Harbor, ME, USA. The mice possessed a GFAP-GFP construct, which allowed for the expression of green fluorescent protein (GFP) under the glia-specific GFAP (Glia fibrillary acid protein) promoter. This allows for the monitoring of GFAP expression and enteric glia proliferation by measuring the amount of fluorescence produced, as explained at length in Section 4.2. Adult breeder mice were maintained on the assigned diet prior to mating, through pregnancy, and nursing time, where food was provided ad libitum. The control diet consisted of a Modified Open Standard Diet with 16 kcal% Fat, Cat# D12020102, while the PPA-rich diet consisted of a Modified Open Standard Diet with 16 kcal% Fat with PPA added at a concentration of 5000 mg/kg of total food, Cat# D19071504. Both diets were acquired from Research Diet Inc., New Brunswick, NJ, USA. The concentration of PPA in the custom diet was chosen, as it corresponds to the highest permitted concentration of PPA and its salts (sodium, potassium, and calcium salts designated as E 281, E 282, and E 283, respectively) in the Europe and the USA as a food additive.

The animals used in this study were humanely sacrificed at either 1-month post-partum (referred as 1 M throughout this study) or at 5 months post-partum (referred as 5 M) by subjecting them to 5% Isoflurane (Cat# 792632-250 MG, Sigma-Aldrich, St. Louis, MO, USA) delivered via a nose cone for 10 min, after which cervical dislocation was performed. The distribution of male and female mice in each group was equal. Each group of 1 M and 5 M was further subdivided into the control group, in which the mice were maintained on the control diet (same as their parents prior to and during pregnancy), and to the PPA-rich diet group, in which the mice and their parents were maintained on the PPA-rich diet. At the time of sacrifice, the body weight of the animals of the 1 M group was between 18 and 22 g, while in the 5 M group it was between 22 and 28 g. The extracted intestine was washed with PBS and subjected to fluorescence measurement from the GFAP-GFP construct, as described in Section 4.2. Intestinal samples were also frozen for subsequent protein concentration analysis (as described in Section 4.5) or preserved with RNAlater (Cat# AM7021, Thermo Fisher Scientific, Waltham, MA, USA) and stored at −20 °C for RNA extraction and RT-qPCR procedures (described in detail in Section 4.4).

### 4.2. The Assesment of the Presence of GFAP Abundance Based on Fluoroscence from the GFAP-GFP Construct

To assure that the assessment of GFAP expression in the intestine (based on the fluorescence from the GFAP-GFAP construct) was performed correctly, the animals included in the study were tested for the presence of the construct with touchdown PCR and gel electrophoresis on the DNA sample obtained from the tail snips. The procedure was performed in accordance with previously published protocol [56]. First, the tail snips were processed with lysis buffer (0.4 mg/mL proteinase K [V3021, Promega, Madison, WI, USA], 10 mM Tris, pH 8.00, 100 mM NaCl, 10 mM Ethylenediaminetetraacetic acid [EDTA], 0.5% Sodium Dodecyl Sulfate [SDS]) overnight in a 56 °C water bath. This was followed by DNA precipitation with 3 M of an NaCl solution, centrifugation, and 70% ethanol wash. After drying in the rotary evaporator (DNA 120 SpeedVac, Thermo Fisher Scientific, Waltham, MA, USA), the DNA was resuspended in TE buffer (10 mM Tris-HCl, pH 8.0, 0.1 mM EDTA, Cat# 12090015, Thermo Fisher Scientific, Waltham, MA, USA). For each PCR reaction, 200 ng of DNA and the following master mix were used: Taq, Cat# M7502, (Promega, Madison, WI, USA). The primers that were used had the following sequence: Forward: 5′ ACT CCT TCA TAA AGC CCT CG 3′ and Reverse: 5′ AAG TCG ATG CCC TTC AGC TC 3′. The following settings were used for the thermocycler (Mastercycler Gradient, Eppendorf, Hamburg, Germany): 95 °C for 6 min, 65 °C for 1.5 min, and 68 °C for 1.5 min. The cycle was then repeated with the annealing temperature decreased by 0.5 °C per cycle until it reached 60 °C, after which another set of cycles (94 °C for 1 min, 65 °C for 1.5 min, and 72 °C for 1.5 min) was repeated 25 times. The resulting PCR product was mixed with a loading dye (Cat# R0611 Thermo Fisher Scientific, Waltham, MA, USA) and ran on a 1.5% agarose gel (with pre-mixed ethidium bromide, Cat# H504, Promega, Madison, WI, USA). The PCR product length was determined by comparison to a FastRuler Low Range DNA Ladder (Cat# SM1103, Thermo Fisher Scientific, Waltham, MA, USA), which was run alongside the PCR samples. The expected PCR product length was 498 bp. Only animals positive for the GFAP-GFP construct were used in the fluorescence-based GFAP measurement.

After the mice were humanely sacrificed, the intestine was extracted and washed with 1 × PBS, after which the excessive liquid was removed. The intestinal samples were not submerged in media, to avoid fluorescence interference from the media. Blank wells were also included to ensure that the fluorescence measurement by the instrument originated from the intestinal samples alone. The fluorescence measurement was obtained with the In Vivo Imaging System (IVIS, Lumina S5, Perkin Elmer, Waltham, MA, USA) in conjunction with the Living Image software, version 4.7.0 (Perkin Elmer, Waltham, MA, USA). All settings of the instrument were constant across all samples.

### 4.3. Cell Culture and In Vitro PPA Treatment

THP1 monocytes (ATCC TIB-202) were acquired from ATTC (Manassas, VA, USA) and maintained as a suspension culture in a T75 flask in a 37 °C humidified incubator (with 5% CO_2_). The RPMI-1640 media used for the culturing (ATCC, Cat# 30-2001) was supplemented with 10% fetal bovine serum (FBS, ATCC, Cat# 30-2020) and 0.05 mM 2-mercaptoethanol. The differentiation of THP1 monocytes into macrophages was conducted in 12-well cell culture plates (Nunclon Delta Surface, Cat# 150628, Nunc A/S, Roskilde, Denmark) with a concentration of 5 × 10^5^ cells/mL. The cells were exposed to 50 ng/mL phorbol 12-myristate 13-acetate (PMA; Sigma Life Science, St. Louis, MO, USA) for 48 h, after which the media was removed and replaced with fresh media (not containing PMA). The cells were treated with 1 mM and 10 mM of sodium propionate (Cat# P5436-100G, Sigma-Aldrich, St. Louis, MO, USA) dissolved in PBS. After 24 h, the supernatant was used for IL-6 measurement with ELISA, and the RNA was extracted to assess *TNF-α* gene expression (described in detail in Section 4.4).

### 4.4. Gene Expression

As described in Section 4.1, the intestinal samples were preserved with RNAlater and stored at −20 °C until the RNA was extracted. The THP-1 cell culture samples were processed immediately and lysed directly in the cell culture plates. The RNA was extracted with the RNeasy Mini Kit (Cat# 74104, QIAGEN GmbH, Hilden, Germany) or TRIzol RNA Isolation Reagents (Cat# 15596026, Thermo Fisher Scientific, Waltham, MA, USA). Before RNA extraction, the intestinal tissue (jejunum) samples were minced with a scalpel and mixed with either a lysis buffer or TRIzol reagent (for RNeasy or TRIzol extraction, respectively), and the tissue was homogenized with a mechanical tissue grinder (Cat# 02-542-10, Fisher Scientific, Hampton, NH, USA). While using the RNeasy method, the manufacturer protocol supplied with the kit was used, whereas TRIzol extraction was performed as follows: the homogenized tissue mixed with the TRIzol reagent was transferred to a 1.5 mL microcentrifuge tube and mixed with chloroform (Serva, Heidelberg, Germany). After centrifugation, the aqueous layer (top) containing RNA was mixed with ice cold 100% isopropanol, incubated for 2 h at −20 °C, and centrifugated for 30 min at 10,000 RCF at 4 °C. The resulting pellet was washed twice with 70% ice cold ethanol, air dried, and resuspended in nuclease-free water pre-treated with diethylpyrocarbonate (DEPC). For cDNA synthesis, a High-Capacity cDNA Reverse Transcription Kit (Cat# 4368814, Applied Biosystems, Waltham, MA, USA) in conjunction with 1 μg of pure RNA was used in each 20 μL reaction (RNA concentration was measured with NanoDrop OneC, Thermo Fisher Scientific, Waltham, MA, USA). Following cDNA synthesis, the samples were diluted (10- to 20-fold) with nuclease-free water, after which qPCR was performed using PowerUp™ SYBR™ Green Master Mix for qPCR (Cat# A25741, Applied Biosystems, Waltham, MA, USA) and QuantStudio 3 Real-Time PCR System (Applied Biosystems, Waltham, MA, USA). The following primers with a proprietary sequence were purchased from Bio-Rad Laboratories (Hercules, CA, USA): *TUBB3*: qMmuCID0018119, *GFAP*: qMmuCID0020163, *IL-6*: qMmuCED0045760, *TNF-α*: qMmuCED0004141 for mice samples and qHsaCED0037461 for THP1 cell culture samples, *IL-10*: qMmuCID0015452, *iNOS*: qMmuCID0023087, and *CD206*: qMmuCID0012670. The housekeeping gene *GAPDH* (qMmuCED0027497 for mice samples and qHsaCED0038674 for THP1 cell culture samples) was used to normalize the gene expression, and data was presented as 2^(−∆CT)^, where ∆CT = CT_target gene_ − CT_reference gene (GAPDH)_ [56].

### 4.5. Protein Concentration

The protein concentration in the intestinal tissue was measured with the aid of ELISA (enzyme-linked immunosorbent assay), where specific protocols provided by the manufacturer were followed for each individual target. In short, the frozen intestinal tissue (jejunum) was minced with a scalpel blade and submerged in a cell lysis buffer containing protease inhibitors (Cat# 9803, Cell Signaling Technology, Danvers, MA, USA), after which they were homogenized with a mechanical tissue grinder. To further assure cell lysis, the tissue was subjected to brief sonification, while the sample was kept on ice to prevent excessive heating (5 s sonification, 50% amplitude, 450 Sonifier Analog Cell Disruptor, Branson, Brookfield, CT, USA). Then, the samples were centrifugated for 10 min at 10,000 RCF in a centrifuge prechilled to 4 °C. The protein concentration in the debris-free supernatant was assessed using a Bradford assay and a bovine serum albumin standard curve (Quick Start™ Bradford Protein Assay Kit, Cat# 5000201, Bio-Rad, Hercules, CA, USA). The normalized protein concentration was used in the commercially available ELISA kit for each target: Tubulin-IIIβ (Cat# LS-F181991, LS-Bio, Lynnwood, WA, USA), GFAP (Cat# EEL098, Thermo Fisher Scientific, Waltham, MA, USA), IL-6 (Cat# KMC0061 for mice samples and CAT# BMS213-2 for THP1 cell culture samples, Thermo Fisher Scientific, Waltham, MA, USA), TNF-α (Cat# BMS607-2HS, Thermo Fisher Scientific, Waltham, MA, USA), and IL-10 (Cat# BMS614, Thermo Fisher Scientific, Waltham, MA, USA).

### 4.6. Statistical Analysis

GraphPad Prism 10.4.1 software for MacOS (GraphPad Software, Boston, MA, USA) was used to perform all statistical analyses and create all graphs. All data was checked for normal distribution with the Shapiro–Wilk normality test. Significance was determined using an unpaired two-tailed t-test (for normally distributed data) and the Mann–Whitney U test (for non-normally distributed data). ANOVA with Dunnett’s multiple comparison test was used in experiments where more than two groups were present. A *p*-value lower than 0.05 (*), 0.01 (**), 0.001 (***), or 0.0001 (****) was considered significant.

## Figures and Tables

**Table 1:** A comparison of the gene expression of glial, neuronal, and inflammatory markers in brain tissue (previously published data) [56]) and intestinal tissue in 1 M and 5 M mice offsprings. The gene expression is presented using the 2^−∆CT^ method, where both mean expression and SD is noted. Ns: not statistically significant.

Gene Name	Time Point	Treatment	Brain (Previously Published Study; 2^−∆CT^)	*p*-Value	Intestine (Current Study; 2^−∆CT^)	*p*-Value
*GFAP*	1 M	Control	0.04492 ± 0.03	<0.05	0.0001614 ± 0.0001	<0.05
		PPA	0.06894 ± 0.03		0.0002490 ± 0.0001	
	5 M	Control	0.04722 ± 0.02	<0.05	0.0001955 ± 0.0001	<0.05
		PPA	0.07712 ± 0.02		0.0003147 ± 0.0001	
*Tubulin IIIβ*	1 M	Control	0.1776 ± 0.03	ns	0.002284 ± 0.001	ns
		PPA	0.1736 ± 0.03		0.002051 ± 0.0002	
	5 M	Control	0.1550 ± 0.05	ns	0.001807 ± 0.001	ns
		PPA	0.1211 ± 0.05		0.001993 ± 0.001	
*IL-6*	1 M	Control	0.001691 ± 0.0006	ns	0.003242 ± 0.002	ns
		PPA	0.002080 ± 0.001		0.002027 ± 0.002	
	5 M	Control	0.0004140 ± 0.0003	<0.01	0.001022 ± 0.0007	<0.05
		PPA	0.001028 ± 0.0005		0.0004502 ± 0.0003	
*TNF-α*	1 M	Control	0.0003728 ± 0.0003	<0.001	0.001872 ± 0.0015	<0.05
		PPA	0.001060 ± 0.0004		0.001054 ± 0.001	
	5 M	Control	0.0001307 ± 0.00008	<0.01	0.001828 ± 0.0016	<0.01
		PPA	0.0003452 ± 0.0002		0.0007326 ± 0.0004	
*IL-10*	1 M	Control	0.0009324 ± 0.0008	ns	0.00001475 ± 0.000009	ns
		PPA	0.0009463 ± 0.0003		0.00001487 ± 0.000009	
	5 M	Control	0.002095 ± 0.0005	<0.01	0.00002411 ± 0.00001	<0.01
		PPA	0.001384 ± 0.0006		0.00003319 ± 0.00001	

## Data Availability

Raw data is available upon request.

## Abbreviations

ASD	autism spectrum disorder
GI	gastrointestinal
DSM-5	Diagnostic and Statistical Manual of Mental Disorders—5th edition
PPA	propionic acid
CNS	central nervous system
ENS	enteric nervous system
AA	acetic acid
BA	butyric acid
SCFA	short chain fatty acid
GFAP	glial fibrillary acidic protein
SD	standard deviation
1 M	1 month
5 M	5 months
ph	photons
sr	steradian
